# Sorption of Salts of Various Metals by Polyelectrolyte Microcapsules

**DOI:** 10.3390/ijms24032834

**Published:** 2023-02-01

**Authors:** Aleksandr L. Kim, Alexey V. Dubrovskii, Egor V. Musin, Sergey A. Tikhonenko

**Affiliations:** Institute of Theoretical and Experimental Biophysics Russian Academy of Science, Institutskaya St., 3, 142290 Puschino, Moscow Region, Russia

**Keywords:** polyelectrolyte microcapsules (PMC), sorption, water treatment, ferrocyanide, ferricyanide, polyallylamine, polystyrene sulfonate

## Abstract

Anthropogenic activity negatively affects the environment by polluting it with the salts of various metals. One of the ways to reduce this influence is to use water purification methods for the salts of various metals. Water purification methods based on nanomaterials are promising. In this regard, we proposed to study polyelectrolyte microcapsules (PMC) as a promising sorption agent for the salts of various metals. It was found that the polystyrene sulfonate-polyallylamine (PSS-PAH) polyelectrolyte complex and polyelectrolyte microcapsules of different compositions are not able to adsorb salts CuSO_4_, Pb(NO)_3_, FeCl_3_, and CuCl_2_. At the same time, it was found that all types of capsules, except for (PSS/PAH)_2_/PSS, are capable of sorbing about 420 µg of K_3_[Fe(CN)_6_] and about 500 µg of K_4_[Fe(CN)_6_] from solution. The adsorption of polyelectrolyte microcapsules has an electrostatic nature which is confirmed by increases in the sorption capacity of PMC of K_3_[Fe(CN)_6_] and K_4_[Fe(CN)_6_] with decreases in the pH of the solution. Also, It was confirmed that the sorption process of PMC of K_3_[Fe(CN)_6_] and K_4_[Fe(CN)_6_] is concentration dependent and has the limitation of the number of binding sites.

## 1. Introduction

The sharp growth in urbanization and industrialization has led to the excessive use of water resources, e.g., rivers, lakes, etc. In particular, such activities lead to water pollution and deterioration of the sanitary state of the environment [1]. One of these pollutants is the compounds of inorganic salts, the presence of which can be associated with both natural and anthropogenic activities [2]. Anthropogenic activity associated with industrial (e.g., fossil fuel combustion, metal processing), agricultural (pesticides), and household activities (e.g., garbage, detergents) [3,4] is a key factor in the increase in the concentration of different metal salts, especially in recent decades [5].

Compounds of various metal salts tend to seep into groundwater and reduce crop yields due to ionic imbalances and cause serious health problems in animals and local communities [6]. In particular, the increased content of ferrocyanide compounds in water leads to an increase in the level of methemoglobin in the blood [7], kidney damage [8], destruction of plant root systems [9], etc. Moreover, compounds of ferricyanides, salts of copper, iron, lead, etc., can lead to the development of muscular and neurological degenerative processes [10], damage to vital organs [11], and a decrease in species diversity and population density of organisms in various ecological niches [12,13]. Therefore, purifying these kinds of pollutants from surface water is necessary.

Wastewater treatment technologies can be divided into traditional and non-traditional methods [14]. Traditional methods include methods such as chemical precipitation, membrane filtration, ion exchange, and electrochemical methods. Each technology has its own advantages and disadvantages. Chemical precipitation is easy to use and low-cost, but the resulting precipitate may be more toxic than the original compound [15,16,17]. Membrane filtration has high pollutant removal efficiency with no need for chemical additives, etc., but is characterized by high costs and labor-intensive manufacturing [15,18,19,20]. The ion exchange method can be used repeatedly and has low costs and high purification efficiency, but at the same time, it is low selective and is not capable of removing low concentrations of substances from the solution [16,17,20,21]. Electrochemical methods are easy to use and eco-friendly but are capital-intensive and inefficient at low contaminant concentrations [15,18]. Due to the shortcomings of the systems described above, research is constantly being conducted to create new ways to purify water of the salts of various metals (non-traditional methods).

Among the non-traditional methods, adsorption methods and methods based on nanomaterials are widely used [22]. Adsorption is one of the best methods for removing a wide range of contaminants from water, including salts of various metals. Among its advantages are high cleaning power, relatively low energy consumption and technical requirements for operation, as well as the ability to avoid large secondary pollution [23]. Adsorbents that can be used include activated carbon [24,25], polymer materials [26], agricultural waste [27,28], magnetic [29], and composite adsorbents [30,31]. Depending on the adsorbent used, this method can have its drawbacks; for example, commercial activated carbon is expensive, quickly contaminated with non-target contaminants, and efficiency decreases significantly after reuse, and in the case of polymeric materials, there is a high temperature and pH dependence.

Nanomaterial-based techniques have received particular attention in recent decades due to their high surface-to-volume ratio and unique electronic, optical, and magnetic properties [32,33]. Low-dimensional structures such as nanocarbon compounds, oxides of one or more metals, oxides of non-metals, magnetic nanoparticles, and nanoclays are most commonly used for cleaning, disinfection, and removal of salts of various metals from water [34,35,36]. All these nanostructures are highly reactive and have a high surface area, and many of them are widely distributed in nature or have inexpensive synthetic processes. In the same way, nanocomplexes, nanoplates, and microspheres with hierarchical nanostructures have been used for wastewater treatment. However, the lack of information on the toxicity, environmental, and health effects of nanomaterials hinders their full use [37].

Thus, the use of water purification methods based on nanomaterials is a promising direction, but it requires more study of the system components. Therefore, we proposed to study polyelectrolyte microcapsules (PMC) as a promising sorption agent for salts of various metals since polyelectrolyte microcapsules and the nanostructural components of their shell have been studied in detail in many works. Particularly, the following properties have been studied: migration of polyelectrolyte layers while CaCO_3_ core dissolving [38]; SEM of PMC at normal conditions [39] and at different ionic strengths and temperatures [40]; destruction of PMC formed on CaCO_3_ microparticles and the release of a protein encapsulated by adsorption [41] and coprecipitation methods [42]; the cytotoxic effect of on macrophages and tumor cells [43]; the mechanical properties of PMC filled with a neutral polymer [44] or protein [45], etc.

## 2. Results and Discussion

**Figure 1:**
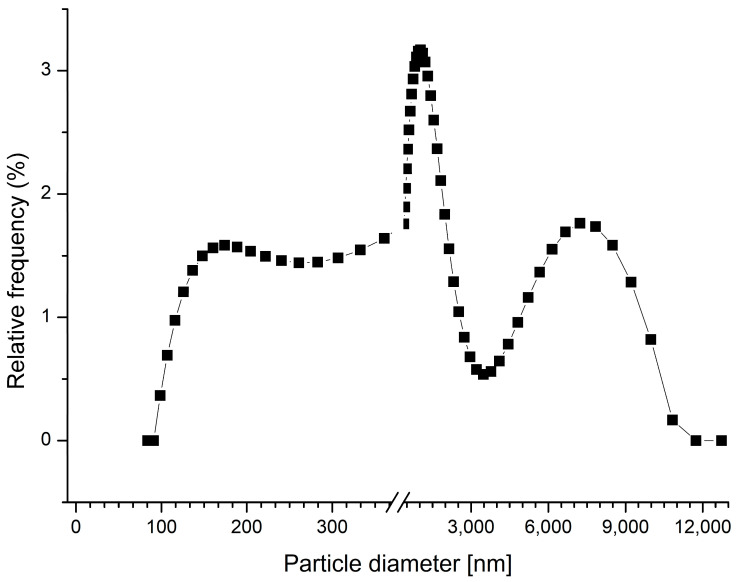
Size distribution of PSS-PAH particles.

As known, polyelectrolyte complexes may be used in the sorption of toxic pollutants [46,47], such as K_4_[Fe(CN)_6_], K_3_[Fe(CN)_6_], CuSO_4_, Pb(NO)_3,_ FeCl_3_, and CuCl_2_, which are widely used or are by-products in the mining extraction and processing industries and in the production of polymeric materials, paints, and varnishes [2]. Therefore, the first step in creating a sorption system was to study the possibility of sorption of those substances using a PSS-PAH polyelectrolyte complex. For that purpose, a PSS-PAH polyelectrolyte complex was formed by mixing two polyelectrolytes in a ratio of 1:1 with constant stirring at 300 rpm. The obtained results are shown in Figure 1.

The size distribution demonstrates the morphological heterogeneity of the PSS-PAH particles in a range of 100–10,000 nm. The hydrodynamic diameter of obtained particles is 904.6 nm with a 34.4% polydispersity index and a 0.54 diffusion coefficient.

The obtained particles were added to a solution of the required salt and incubated with salt for 10 min. The results obtained are presented in Table 1.

Table 1 shows a significant decrease in the concentrations of solutions of salts of K_4_[Fe(CN)_6_] (at 319 nm) and K_3_[Fe(CN)_6_] (at 422 nm). This effect may be associated with the formation of ionic bridges between FCIV/FCIII ([Fe-(CN)_6_]^4−^/[Fe(CN)_6_]^3−^) and several molecules of polyallylamine [48,49]. In addition, upon incubation of a PAH solution in a solution with salts K_4_[Fe(CN)_6_] and K_3_[Fe(CN)_6_], turbidity of the solution and precipitation was observed, which is also associated with the formation of a complex between PAH and [Fe-(CN)_6_]^4−^/[Fe(CN)_6_]^3−^ ions. Upon incubation of the PSS-PAH polyelectrolyte complex with other salts, no similar effect was observed.

**Figure 2:**
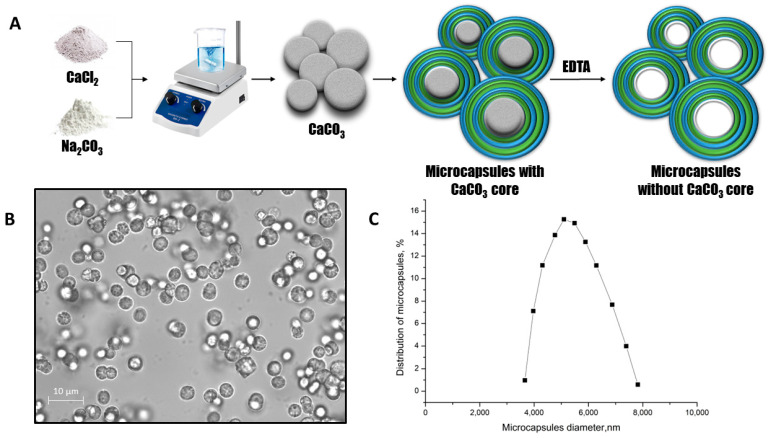
The scheme of the polyelectrolyte microcapsules preparation (**A**). The optical microscopy images of PMC (**B**). The PMC diameter distribution function (**C**).

As shown above, the PSS-PAH polyelectrolyte complex was able to bind with salt compounds effectively, but the extraction of the complex requires additional labor costs, or these complexes must be fixed on the surface, which will result in a significant decrease in their efficiency due to a surface area reduction. Polyelectrolyte microcapsules based on these polyelectrolytes are a more suitable sorption agent because they are easier to remove from the medium [50]. Therefore, we prepared polyelectrolyte microcapsules by layer-by-layer adsorption of polyelectrolytes polystyrene sulfonate (PSS) and polyallylamine (PAH) on the CaCO_3_ particle with the later dissolution of it. A main scheme of the preparation of the polyelectrolyte microcapsules containing peroxidase is shown in Figure 2A.

The optical microscopy images of PMC (Figure 2B) demonstrate the morphological homogeneity of microcapsules. The average diameter of microcapsules containing peroxidase was 5.1 μm with a 22.6% polydispersity index (Figure 2C), and the microcapsule’s ζ-potential is +25 ± 2 mV.

The next step was to determine the sorption capacity of polyelectrolyte microcapsules, the shell of which consisted of PSS and PAH polyelectrolytes, but with a different number and sequence of layering of polyelectrolyte layers. The polyelectrolyte microcapsules with different compositions may have different sorption capacities due to the different physicochemical properties, for example, buffer capacity, dissociation level, protonation level, etc. [41,51]. The obtained microcapsules had the compositions (PSS/PAH)_3_, (PAH/PSS)_3,_ (PSS/PAH)_3_PSS, or (PAH/PSS)_3_PAH. The PMC composition, for example (PSS/PAH)_3_, means that polystyrene was used as the first layer in the preparation of the microcapsules’ shell, further layers were applied alternately up to the sixth layer, and the polyallylamine layer was the last one. The results obtained are presented in Table 2.

As can be seen from Table 2, in the case of CuSO_4_, Pb(NO)_3_, and CuCl_2_, the sorption of PMC of any composition does not exceed 3–4%, which is an unreliable result and is within the statistical error, so the amount of sorbed substance in Table 2 is 0. In the case of incubation of capsules in FeCl_3_ solution, we observe an increase in optical density, which is associated with precipitation. Presumably, this is due to the formation of iron hydroxide as a result of the reaction between ferric chloride and calcium carbonate that was not completely removed from the PMC core.

**Figure 3:**
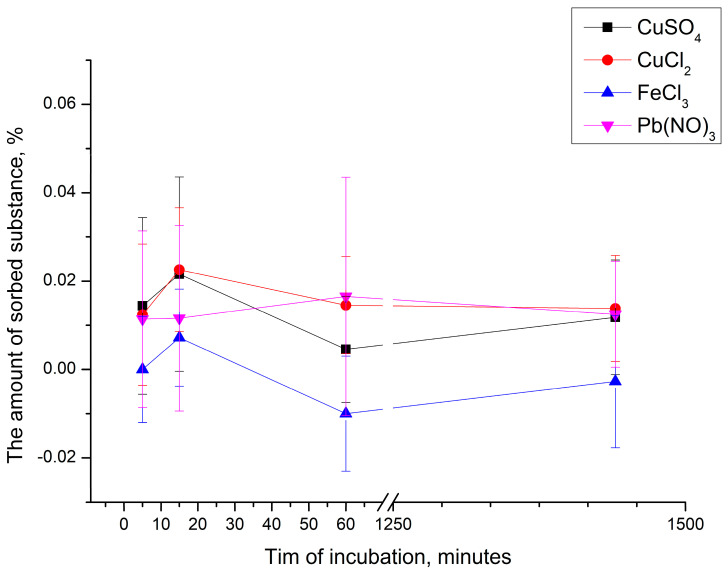
Change in the amount of sorbed of CuSO_4_, Pb(NO)_3,_ FeCl_3_, and CuCl_2_ by PMC depending on the time of incubation.

Subsequently, the polyelectrolyte microcapsules described above were incubated for 24 h in the appropriate salt solutions to confirm the absence of sorption over time. The results obtained are presented in Figure 3.

As can be seen from Figure 3, the amount of sorbed substance does not exceed 1% even after 24 h of incubation. Thus, we can conclude that polyelectrolyte microcapsules consisting of PSS and PAH are not suitable for the sorption of CuSO_4_, Pb(NO)_3,_ FeCl_3_, and CuCl_2_ salts.

At the same time, as can be seen from Table 2, all types of capsules, except for (PSS/PAH)_2_/PSS, are capable of sorbing about 420 µg of K_3_[Fe(CN)_6_] from the solution. As in the case of PSS-PAH complexes, this can be related to the formation of ionic bridges between FCIV/FCIII ([Fe-(CN)_6_]^4−^/[Fe(CN)_6_]^3−^) and amino groups of polyallylamine molecules. PMC of composition (PSS/PAH)_2_/PSS adsorbs only about 209 µg K_3_[Fe(CN)_6_]. Perhaps this is due to the fact that most of the positively charged amino groups of PAH are compensated by oppositely charged groups of polystyrene sulfonate [51,52,53,54]. This results in the formation of weak ionic bridges between [Fe(CN)_6_]^3−^ and PAH, which are not enough to retain the salt in the capsule. In the case of K_4_[Fe(CN)_6_] sorption by capsules of various compositions, it can be seen that the sorption capacity is about 500 µg. This is slightly higher than in the case of K_3_[Fe(CN)_6_] and corresponds to the data obtained above for PSS-PAH complexes.

**Figure 4:**
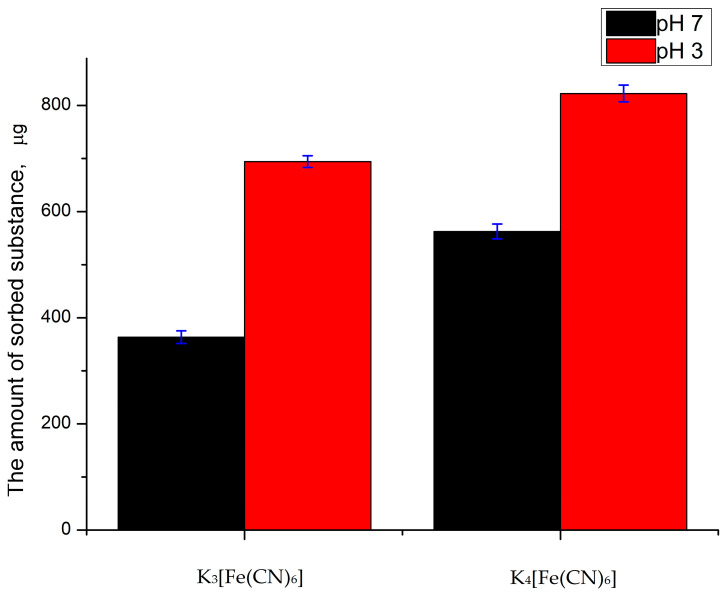
PMC’s (PSS/PAH)_3_ sorption capacity of solutions of K_3_[Fe(CN)_6_] and K_4_[Fe(CN)_6_] at pH 7 and 3.

To confirm the electrostatic nature of the adsorption of FCIV/FCIII salts ([Fe-(CN)_6_]^4−^/[Fe(CN)_6_]^3−^), the effect of a more acidic environment on the sorption capacity of capsules was studied. According to the hypothesis, a decrease in the pH of the solution will lead to an increase in the number of amino groups of PAH with a positive charge (it is a weak polyelectrolyte) [55,56]. This, in turn, will increase the number of bound K_3_[Fe(CN)_6_] and K_4_[Fe(CN)_6_] molecules. For that purpose, we incubated (PSS/PAH)_3_ microcapsules in the K_3_[Fe(CN)_6_] and K_4_[Fe(CN)_6_] solutions with pH 3 or 7 for 10 min and determined the concentration of the substance after sedimentation of the capsule. The results obtained are shown in Figure 4.

As can be seen from the figure, as the pH of the solution decreases, the sorption capacity of PMC of K_3_[Fe(CN)_6_] and K_4_[Fe(CN)_6_] salts increases. This confirms the hypothesis described above about the electrostatic nature of the adsorption of polyelectrolyte microcapsules.

**Figure 5:**
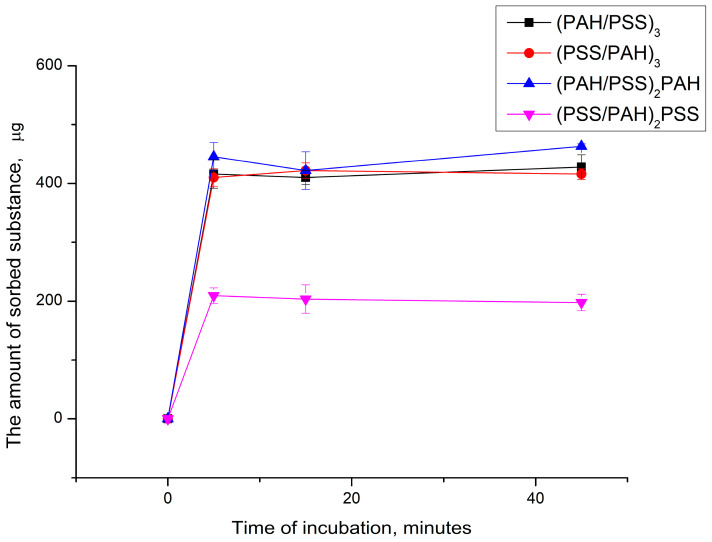
Change in the amount of sorbed potassium ferricyanide by PMC depending on the time of incubation.

The adsorption of PMC salts of K_3_[Fe(CN)_6_] and K_4_[Fe(CN)_6_] has the same nature; for that reason, the dynamics of sorption of only K_3_[Fe(CN)_6_] by polyelectrolyte microcapsules was further studied. For that purpose, the PMCs were obtained with polyelectrolyte layers PSS and PAH but with a different number and order of that layers. The choice is based on the fact that the spectrophotometric method for determining the concentration of K_3_[Fe(CN)_6_] in a solution is more sensitive than for determining the K_4_[Fe(CN)_6_]. The results obtained are shown in Figure 5.

As can be seen from the figure, the amount of the sorbed substance does not change with increasing incubation time. Perhaps this is due to the fact that the adsorption of K_3_[Fe(CN)_6_] by polyelectrolyte microcapsules is a concentration-dependent process.

**Figure 6:**
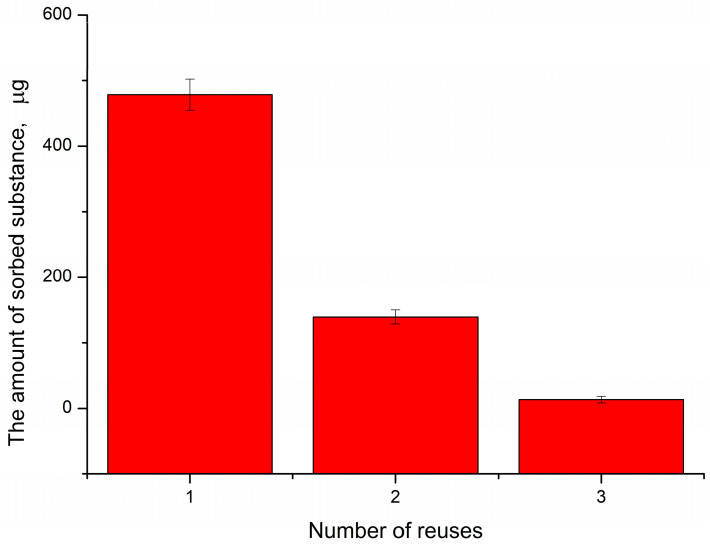
Change in the amount of adsorbed K_3_[Fe(CN)_6_], depending on the number of repeated incubations with PMC.

To test this hypothesis, we incubated PMC of the composition (PSS/PAH)_3_ in 1 mM K_3_[Fe(CN)_6_] solution for 10 min, centrifuged the capsules, discarded the supernatant and filled in a new solution with a concentration of 1 mM, and re-incubated for 10 min with the same PMC. The results obtained are shown in Figure 6.

As can be seen from Figure 6, with repeated use of PMC, the amount of adsorbed K_3_[Fe(CN)_6_] decreased by 20% compared with the first application. Moreover, at the third use, the adsorption of K_3_[Fe(CN)_6_] by capsules was absent. This result allows us to conclude that this sorption process is concentration-dependent, but at the same time, it has the limitation of the number of PMC shell binding sites with K_3_[Fe(CN)_6_]. This corresponds to the literature data on the formation of an ionic bond between the PAH/Polyglutamic acid polyelectrolyte complex and [Fe-(CN)_6_]^4−^/[Fe(CN)_6_]^3−^ [48,49] because a local increase in the concentration of K_3_[Fe(CN)_6_] inside PMC leads to the substitution of the counterion of the amino group of PAH.

As a result of this study, it was determined that polyelectrolyte microcapsules of the composition (PAH/PSS)_3_, (PSS/PAH)_3_, and (PAH/PSS)_2_/PAH are capable of sorbing more than 420 µg K_3_[Fe(CN)_6_] and K_4_[Fe(CN)_6_] from solution, while the sorption process is concentration-dependent and has the limitation of the number of binding sites. This is due to the formation of ionic bonds between [Fe(CN)_6_]^4−^/[Fe(CN)_6_]^3−^ and PAH amino groups.

## 3. Materials and Methods

### 3.1. Materials

Polyelectrolytes polystyrenesulfonate sodium (PSS) and polyallylamine hydrochloride (PAH) with a molecular mass of 70 kDa and ethylenediaminetetraacetic acid disodium salt dihydrate (EDTA) were purchased from Sigma (St. Louis, MO, USA). Sodium chloride, sodium sulfate, sodium carbonate, calcium chloride, potassium ferricyanide, potassium ferrocyanide, copper chloride, copper sulfate, iron(III) chloride, and lead(II) nitrate were purchased from “Reahim”(Moscow, Russia).

### 3.2. Preparation of CaCO_3_ Microspherulites

A total of 0.33 M Na_2_CO_3_ was added to 0.33 M CaCl_2_ and stirred for 30 s. The suspension was maintained until complete precipitation of the formed particles. The process of “ripening” the microspherolites was controlled with the help of a light microscope. Then, the supernatant was decanted, and the precipitate was washed with water and used to prepare PMC. The microparticles obtained had an average diameter of 5 ± 2 μm.

### 3.3. Preparation of Polyelectrolyte Microcapsules

The polyelectrolyte microcapsules were obtained by layer-by-layer adsorbing the negatively or positively charged polyelectrolytes onto CaCO_3_ microspherulites, followed by the dissolution of CaCO_3_. At the moment of dissolution of the CaCO_3_ core, the inner space of PMC is filled by an interpolyelectrolyte complex [57]. Layer-by-layer adsorption of PAH and PSS on the CaCO_3_ microspherulites surface was carried out in polyelectrolytes solutions (concentration 2 mg/mL + 0.5 M NaCl). After each adsorption, the CaCO_3_ particles with adsorbed polyelectrolytes were triple-washed with a 0.5 M NaCl solution, which was necessary to remove unadsorbed polymer molecules. The particles were separated from the supernatant with centrifugation. After applying the required number of layers, the carbonate kernels were dissolved in a 0.2 M EDTA solution for 12 h. The resulting capsules were washed three times with water to remove core decay products. The microcapsules obtained had an average diameter of 5.1 ± 2 μm. The size and number of microcapsules were measured using the dynamic light scattering method on a Zetasizer nano ZS device (Malvern, UK).

### 3.4. Measurement of Sorption Capacity of PMC

Polyelectrolyte microcapsules were added to 2 mL of salt solution in the amount of 8 × 10^8^ pieces and incubated on a shaker for 10 min. After that, the PMC suspension was precipitated in a centrifuge at 10,000× *g*. Subsequently, the supernatant was transferred to a quartz cuvette, and the optical density of the solution was measured at a certain wavelength according to the salt used.

### 3.5. Statistical Processing

Each sample was measured five times and was calculated as average and standard deviation.

## 4. Conclusions

As a result of this study, it was found that the PSS-PAH polyelectrolyte complex and polyelectrolyte microcapsules of different compositions are not able to adsorb salts CuSO_4_, Pb(NO)_3_, FeCl_3_, and CuCl_2_.

At the same time, it was found that all types of capsules, except for (PAH/PSS)_3_, (PSS/PAH)_3_, and (PAH/PSS)_2_/PAH are capable of sorbing about 420 µg of K_3_[Fe(CN)_6_] from the solution. PMC of composition (PSS/PAH)2/PSS adsorbs only about 209 µg K_3_[Fe(CN)_6_]. This can be related to the formation of ionic bridges between FCIV/FCIII ([Fe-(CN)_6_]^4−^/[Fe(CN)_6_]^3−^) and amino groups of polyallylamine molecules. That hypothesis was confirmed by increases in the sorption capacity of PMC of K_3_[Fe(CN)_6_] and K_4_[Fe(CN)_6_] in the solution with a low pH since at low pH, the weak polyelectrolyte PAH has more protonated sites.

Moreover, the electrostatic nature of PMC’s sorption is confirmed by the concentration-dependent sorption by PMC of K_3_[Fe(CN)_6_] and K_4_[Fe(CN)_6_] and by the limitation of the number of binding sites.

## Figures and Tables

**Table 1:** Concentration of salts of various metals before and after incubation of the polyelectrolyte complex PSS-PAH in solution.

	K_3_[Fe(CN)_6_]	K_4_[Fe(CN)_6_]	CuSO_4_	Pb(NO)_3_	FeCl_3_	CuCl_2_
Concentration before incubation	1 mM	5 mM	100 mM	143 mM	0.625 mM	100 mM
Concentration after incubation	0.356 mM	0.22 mM	100 mM	143 mM	0.625 mM	100 mM

**Table 2:** The amount of adsorbate (salts of various metals) taken up by the various type of PMC.

PMC’s Type	K_3_[Fe(CN)_6_], µg	K_4_[Fe(CN)_6_], µg	CuSO_4_, µg	Pb(NO)_3,_ µg	FeCl_3,_ µg	CuCl_2,_ µg
(PAH/PSS)_3_	428.1726	459.5166	0	0	−31.1923	0
(PSS/PAH)_3_	422.2668	568.4712	0	0	−48.5214	0
(PAH/PSS)_2_/PAH	445.8901	515.1191	0	0	−51.9872	0
(PSS/PAH)_2_/PSS	209.657	382.4399	0	0	−41.5897	0

## Data Availability

Not applicable.
